# Obstetric infections and clinical characteristics of maternal sepsis: a hospital-based retrospective cohort study

**DOI:** 10.1038/s41598-024-56486-4

**Published:** 2024-03-13

**Authors:** Sedina Atic Kvalvik, Sofie Branæs Zakariassen, Sofie Overrein, Svein Rasmussen, Steinar Skrede, Elham Baghestan

**Affiliations:** 1 Department of Obstetrics and Gynaecology, Haukeland University Hospital, Pb 1400, 5021 Bergen, Norway; 2 Department of Clinical Science, University of Bergen, Pb 7804, 5020 Bergen, Norway; 3 Department of Medicine, Haukeland University Hospital, Pb 1400, 5021 Bergen, Norway

**Keywords:** Diseases, Infectious diseases, Reproductive signs and symptoms, Fever, Epidemiology, Health care

## Abstract

Sepsis is responsible for 50% of intrahospital maternal deaths worldwide. Incidence is increasing in both low and middle-, and high-income countries. There is little data on incidence and clinical outcomes of obstetric infections including maternal sepsis in the Nordic countries. The aims of this study are to give estimates of the occurrence of obstetric infections and maternal sepsis in a Norwegian hospital cohort, assess the quality of management of maternal sepsis cases, and evaluate the usefulness of diagnostic codes to identify maternal sepsis retrospectively. We conducted a retrospective cohort study of pregnant, labouring, post-abortion, and postpartum women. We assessed the accuracy of the diagnostic code most frequently applied for maternal sepsis, O85. We found 7.8% (95% confidence interval 7.1–8.5) infection amongst pregnant, labouring, and postpartum women. The incidence of maternal sepsis was 0.3% (95% confidence interval 0.2–0.5), and the majority of sepsis cases were recorded in the postpartum period. Two thirds of women were given broad-spectrum antibiotics at the time sepsis was diagnosed, but only 15.4% of women with puerperal sepsis were given antimicrobials in accordance with national guidelines. When used retrospectively, obstetric infection codes are insufficient in identifying both maternal and puerperal sepsis, with only 20.3% positive predictive value for both conditions. In conclusion, obstetric infections contribute significantly to maternal morbidity in Norway’s second largest maternity hospital. This study provides incidences of maternal infections for hospitalised patients in temporal relation to pregnancy, labour, abortion and the postpartum period, knowledge which is valuable for planning of health care services and allocation of resources. In addition, the study highlights areas where improvement is needed in clinical handling of maternal sepsis. There is need for studies on the management quality and use of correct diagnostic codes in this patient category.

## Introduction

Maternal sepsis is considered the most severe infection related to childbirth and is globally ranged as one of the five leading causes of maternal mortality^1^. In 2020 the World Health Organization (WHO) stated that almost half of intrahospital maternal deaths worldwide are caused by infection and sepsis, suggesting that the impact and burden of obstetric infections previously have been underestimated^2^. The last two decades increasing trends in maternal infectious morbidity and mortality, predominantly in low- and middle-income countries, have been reported^3^. Similar trends are seen in high-income countries^4,5^.

Obstetric infections constitute both incidental and pregnancy specific infections which can occur during pregnancy, in labour, post labour or after abortion^6^. The association of infection in temporal relation to pregnancy, labour and postpartum period is incompletely known, but reportedly more than two thirds of the infections occur postpartum^7^.

Partly due to their unique physiology requiring different reference values to describe normality, obstetric patients are, however, excluded from the sepsis definitions for the general population^8–10^. WHO stated in 2017 that there is an urgent need for a generally accepted definition of maternal sepsis^11^, and recently consensus has been achieved defining the condition^12^.

Despite the serious potential outcome of obstetric infectious morbidity, there is neither a surveillance program nor a registry of maternal sepsis in Norway. Furthermore, there is no specific diagnostic code for maternal sepsis, and the relevant diagnostic codes in the International Statistical Classification of Disease and Health Related Problems, Tenth Edition (ICD-10) are not evaluated in their precision to encode the condition.

Consequently, our knowledge on the women who survive obstetric infection is limited^13,14^. A report from 2014 based on an audit on maternal deaths in Norway in the years 1996–2011 concluded that substandard care was given in two thirds of cases and that earlier recognition and more appropriate management of sepsis could have prevented the maternal deaths^15^. There are 70 survivors per maternal death caused by sepsis, and studies show that also in maternal near-miss sepsis cases, clinical handling needs improvement^16^. There is need for data on incidence and outcomes of obstetric infections, and maternal sepsis in particular, in accordance with WHO’s focus on obstetric infections in the twenty-first century^11^.

The present study aimed to examine the occurrence of obstetric infections in a Norwegian hospital cohort and offer an estimate of the contemporary incidence of maternal sepsis. We also wanted to evaluate the accuracy in the use of obstetric diagnostic codes for infection, as well as the codes’ ability to identify cases with maternal sepsis when used retrospectively. Finally, we wished to evaluate whether the clinical management was in accordance with the established sepsis guidelines for the general population^17–19^.

## Methods

We conducted a retrospective cohort study of three subgroups of women; the first and second categories being women undergoing either legal or spontaneous abortion, and the third represented by women ≥ 16 weeks pregnant, labouring women, and postpartum women at the women’s clinic, Haukeland University Hospital (HUH) between 1 January and 31 December 2016. This particular year was chosen to create sufficient time distance to the implementation of an obstetrically modified early warning score system in 2018, and thus ensure that the results were unaffected by this. HUH is a tertiary care referral centre and university hospital with a catchment area of 380,000 individuals, and with approximately 5000 births annually it is the second largest maternity hospital in Norway. Both inpatient and outpatient women registered with obstetric infection after legal and spontaneous abortion, during pregnancy, in labour or up to 42 days postpartum, i.e., the puerperium, were included. Pregnant and postpartum women seeking primary or private care due to obstetric infections were not included in our study unless they were referred to our hospital.

**Figure 1:**
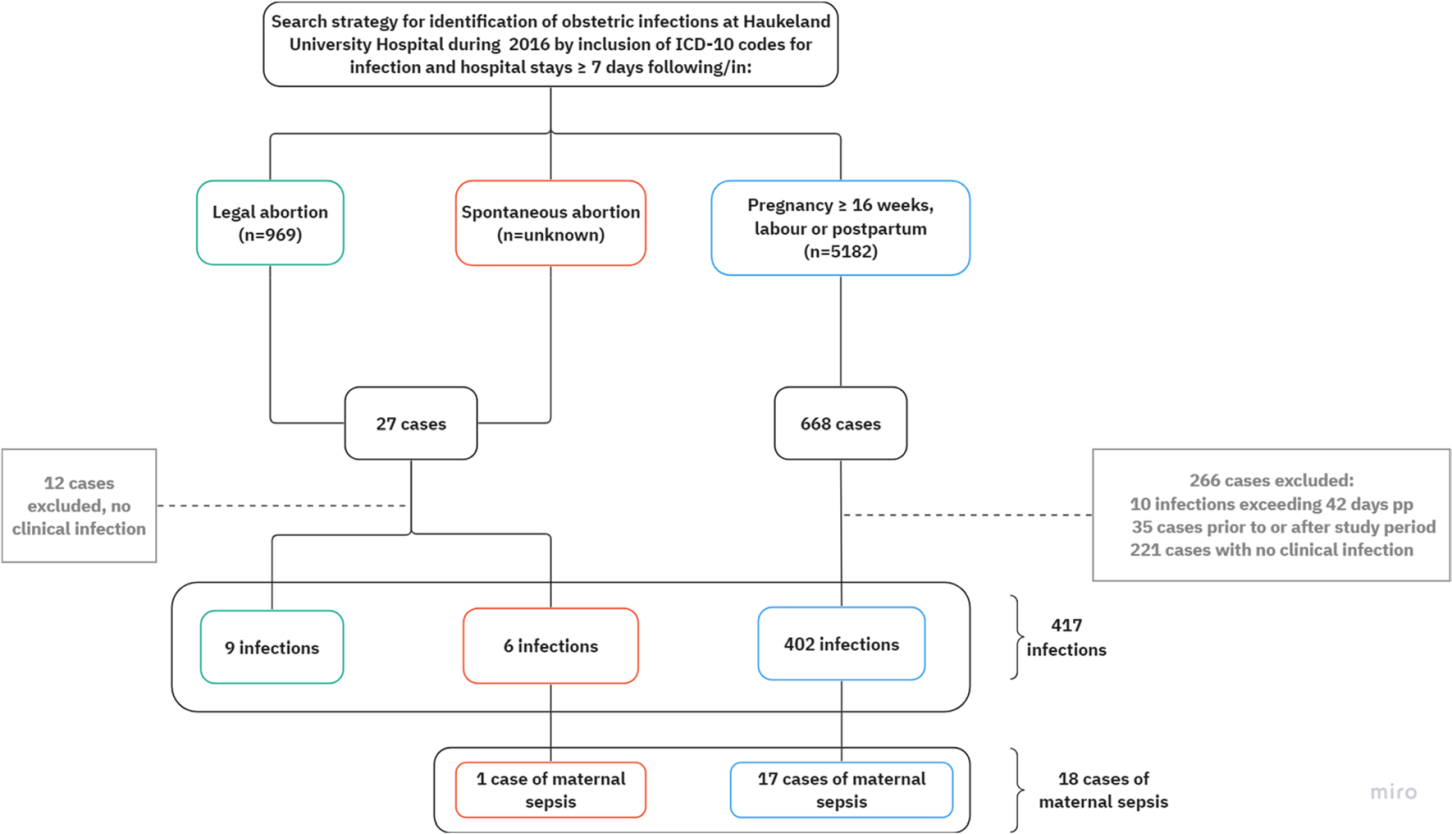
Flow chart of search strategy for obstetric infection codes and hospital stays 7 days or longer in the pregnant, labouring, post-abortion, and postpartum population at Haukeland University Hospital January to December 2016. Green represents infections following legal abortion, red represents infections following spontaneous abortion and blue represents infections following infections in pregnancy ≥ 16 weeks, in labour or up to 6 weeks postpartum. Abbreviation: pp; postpartum. Figure created with software from Miro© (2024), version 2.0, Miro.com.

Cases with infection were identified by a search for obstetric infection codes (O-codes) after legal and spontaneous abortion and during pregnancy, labour and postpartum according to the International Statistical Classification of Disease and Health Related Problems, Tenth Edition (ICD-10)^20^. Selected diagnostic codes and instruction for obstetric sepsis registration are presented in Table S1 (Supplementary information). Data were obtained from the electronic patient medical record systems. Clinical infections as recorded in the medical records were considered to represent the “gold standard” in quality assessment of the diagnoses. Additionally, we included patients admitted to the maternity department or readmitted after labour for seven days or more to detect infections that possibly were not encoded with any of the codes listed in Table S1 (Supplementary information). Women who had given birth outside the study period or were transferred from other hospitals after giving birth, or who developed infection more than 42 days postpartum as well as women without infection, were excluded. Flow chart of the search strategy is presented in Fig. 1. One diagnostic code of infection represented one case of infection, and one woman could have had more than one infectious episode.

Clinical data included respiration rate, peripheral oxygen saturation, body temperature, blood pressure, heart rate, and mental state. Fever was defined as body temperature 38 °C or higher. Clinical and laboratory findings (leukocytes and microbiological findings in cultures where available) were registered for each patient. We did not register whether patients were given antibiotic prophylaxis during labour. However, antibiotic prophylaxis is routinely given in emergency caesarean section cases in accordance with the Norwegian national guideline for intrahospital antibiotic treatment and the WHO^21,22^. Medical records were reviewed by three experienced doctors, and for each patient it was concluded whether there was compliance between the diagnostic codes used and clinical infection category. Where both symptoms and clinical infection signs were present, for instance fever in combination with abdominal pain, uterine tenderness on palpation, foul smelling lochia, swelling and/or redness of breasts, surgical sites or perineal or vaginal tears, in addition to findings in laboratory tests (elevated inflammation markers as mentioned above and/or positive bacterial cultures), we concluded that there was an infection. Where infection was confirmed, we concluded its focus based on the description in the medical journals. To define the presence of chorioamnionitis, we applied the clinical criteria suggested by the National Institute of Child Health and Human Development (NICHD) Workshop expert panel^23^. Our primary treatment method of legal abortion consists of a combination of orally administered mifepristone followed by orally, then transvaginally administered misoprostol. We treat post-abortion infections surgically with evacuation of the uterus, in combination with antimicrobial therapy.

### Assessment of maternal sepsis

Maternal sepsis was categorized according to the WHO criteria and defined as a *life-threatening condition with organ dysfunction resulting from infection during pregnancy, childbirth, post-abortion, or postpartum period*^12^. The definition is based upon vital parameters as described in the SEPSIS-3 for the adult population while taking into consideration pregnant physiology by applying obstetrically modified score systems, as well as clinical and laboratory findings^8,12^. In cases with ambiguity whether sepsis was present or not, a specialist in infectious diseases reviewed and categorized these. For women fulfilling the sepsis definition, maternal age, pre-gestational body mass index (BMI), parity, tobacco use in the first trimester, mode of delivery, duration of labour, number of vaginal examinations during labour, induction of labour, estimated blood loss, and length of hospital stay were recorded. Timing of sepsis in relation to pregnancy, labour, post-abortion, or postpartum period, presence of pathogens in cultures, and values for physiological parameters at the time of sepsis diagnosis were determined. We further recorded whether sepsis treatment was initiated at the time of diagnosis, and if selection of empiric antibiotic treatment adhered to the national guideline. For puerperal sepsis a combination of penicillin, gentamicin and clindamycin is recommended^21^. We further registered whether source control with drainage of potential infected foci was undertaken, i.e., evacuation of retained products from the uterus in case of endometritis, and drainage of abscesses in case of mastitis, intraabdominal-, perineal- or vaginal infection.

### Statistical analyses

We assessed the quality of the relevant diagnostic codes (predominantly O85) by calculating the sensitivity, specificity, positive and negative predictive values. Statistical analyses were performed with SPSS (IBM Corp. Released 2019. IBM SPSS Statistics for Windows, Version 26.0. Armonk, NY: IBM Corp). To calculate confidence intervals for proportions we used the continuity corrected score method^24^.

### Ethical approval

The study was approved by the head of the Women’s clinic at HUH. The requirement for informed consent from the study subjects was waived by the Regional Ethics Committee Health Region West (REC-West number 2019/32278), as the ethical committee classified the study as a quality improvement project. All methods were performed in accordance with the ethical standards of the institutional and national research committee as well as with the Helsinki declaration and its later amendments or comparable ethical standards.

## Results

In this study, we present three different cohorts with separate denominators, and occurrence of infection are therefore given separately after legal—and spontaneous abortion, and in pregnancy, labour, and postpartum period. Altogether, 27 cases fulfilled the search criteria for post-abortion infection, of which 12 were excluded as they did not represent infection (Fig. 1). There were 969 legal abortions registered in the study period, and nine infectious episodes were identified, equalling an occurrence of 0.9% (9/969) (Table 1). There were six infections following spontaneous abortion, however the occurrence of infection after spontaneous abortion remains unknown as the denominator of spontaneous abortions is unexplored in the current study (Table 1).

**Table 1:** Post-abortion infections after legal and spontaneous abortion (n = 15) January to December 2016, Haukeland University Hospital, Norway.

Infection category	Number (%)	Maternal sepsis	Surgical evacuation^a^
Post-spontaneous abortion	6/NA	1/NA	5/6
Post-legal abortion	9/969 (0.9)	0/969	8/9

*NA* not applicable. The denominator of spontaneous abortions is unexplored in the study.

^a^Surgical evacuation as primary mode of treatment in cases of post-abortion infection.

In total there were 5182 deliveries in the study period. Altogether 668 cases fulfilling search criteria were identified, of which 45 cases were excluded because they either delivered outside the inclusion period or at another hospital, or they were registered with infection exceeding 42 days of puerperium, and 221 cases were excluded due to no infection. Most of these were women with hospital stays seven days or longer for other reasons than obstetric infection (Fig. 1). We identified 402 infections in the pregnant, labouring, and postpartum cohort, giving a total frequency of obstetric infection of 7.8% (402/5182, 95% CI 7.1–8.5) (Table 2).

**Table 2:** Infections (n = 402) in the pregnant, labouring, and postpartum population (n = 5182), January to December 2016, Haukeland University hospital, Norway.

Level of care	Inpatient	179 (44.5%)
	Outpatient	223 (55.5%)
Infection category	Number	% (95% CI)
Antepartum urinary tract infection	110	2.1 (1.8–2.6)
Mastitis	83	1.6 (1.6–2.0)
Chorioamnionitis	53	1.0 (0.8–1.3)
Endometritis	49	1.0 (0.7–1.3)
Postpartum urinary tract infection	44	0.9 (0.6–1.1)
Perineal and abdominal wound infection	34	0.7 (0.5–0.9)
Maternal sepsis	17	0.3 (0.2–0.5)
Peritonitis	3	0.1 (0.02–0.2)
Other and unknown infection	9	0.2 (0.1–0.3)
In total	402	7.8 (7.1–8.5)

*CI* confidence interval.

**Figure 2:**
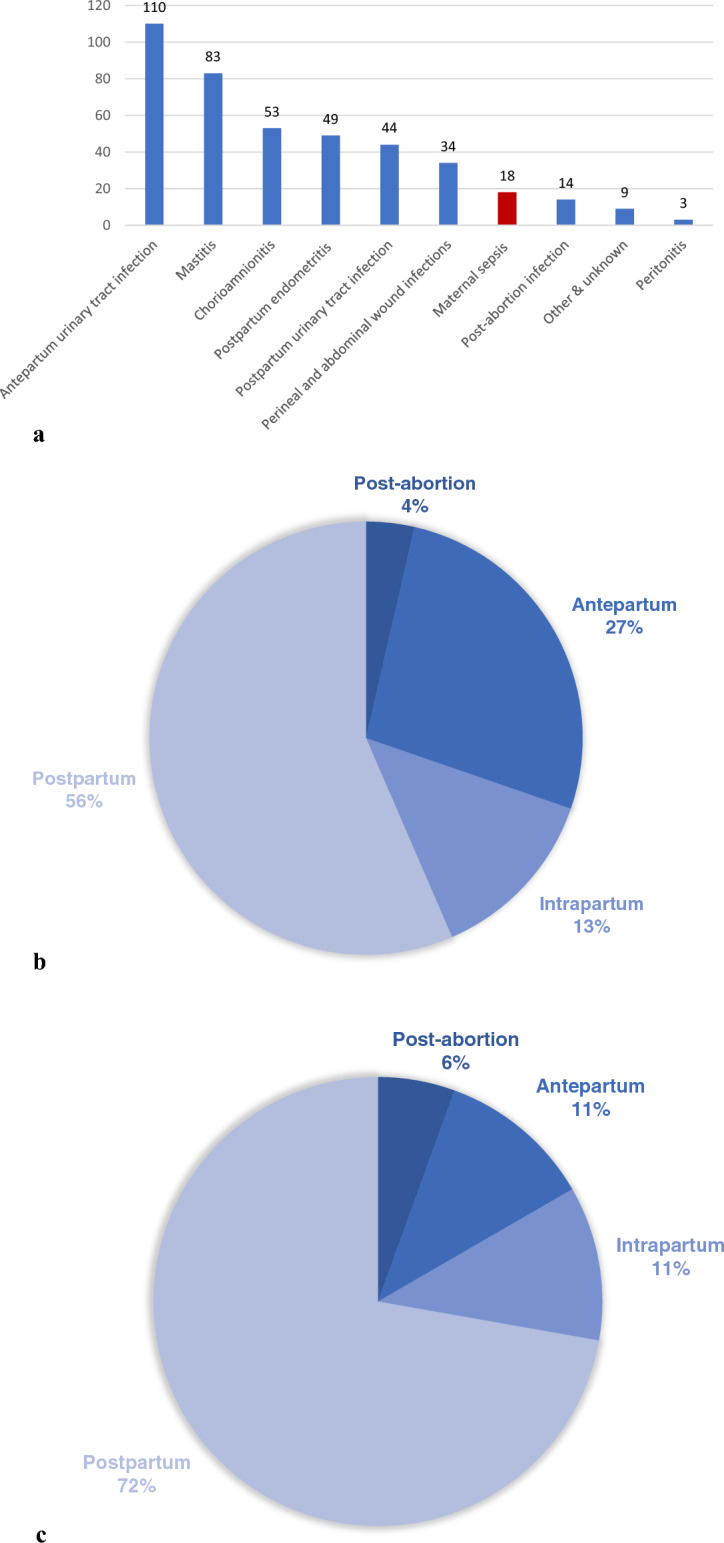
(**a**) Clinical categories in declining order among women with infection at Haukeland University Hospital, January to December 2016 (n = 417). (**b**) Infection and temporal relation to pregnancy, labour, post-abortion, and postpartum period, Haukeland University Hospital, January to December 2016 (n = 417). (**c**) Maternal sepsis and temporal relation to pregnancy, labour, post-abortion, and postpartum period, Haukeland University Hospital, January to December 2016 (n = 18).

The distribution of categories is shown for the entire cohort consisting of 417 infections in Table 3 and Fig. 2a.

**Table 3:** The distribution of infections (n = 417) in the post-abortion and obstetric population, January to December 2016, Haukeland University hospital, Norway.

Level of care	Inpatient	189 (45.3)
	Outpatient	228 (54.7)
Infection category	Number	% (95% CI)
Antepartum urinary tract infection	110	26.4 (22.4–30.8)
Mastitis	83	19.9 (16.4–24.0)
Chorioamnionitis	53	12.7 (9.9–16.3)
Endometritis	49	11.8 (9.00–15.2)
Postpartum urinary tract infection	44	10.6 (8.0–13.9)
Perineal and abdominal wound infection	34	8.2 (5.9–11.2)
Maternal sepsis	18	4.3 (2.8–6.7)
Peritonitis	3	0.7 (0.2–2.1)
Post-abortion infection	14	3.4 (2.0–5.6)
Other and unknown infection	9	2.2 (1.14–4.1)
In total	417	100

*CI* confidence interval.

Most pregnancy related infections were recorded in the postpartum period and included mastitis, endometritis, perineal and abdominal wound infections, in addition to postpartum urinary tract infection, constituting 56% (234/417) (Table 3 and Fig. 2b).

In Table 4, the eighteen cases of maternal sepsis that were identified, are presented. Seventeen cases resulted from pregnancy, labour and the postpartum period, equivalent to an occurrence in that population of 0.3% (17/5182, 95% CI 0.2–0.5). A single case resulted from spontaneous abortion. Thirteen cases were puerperal, accounting for 72.2% of sepsis cases (Fig. 2c). In the postpartum period endometritis was most frequent and recorded in seven sepsis cases, accounting for 38.9%. At the time of sepsis diagnosis, most women had an aberration in vital parameters. There were no women with affection of the central nervous system i.e., reduced consciousness at the time sepsis was diagnosed.

**Table 4:** Pregnancy, labour, and clinical characteristics in the maternal sepsis cohort (n = 18), January to December 2016, Haukeland University Hospital.

Characteristic	Category	Number (%)
Age	≤ 25.0	3 (16.7)
	25.1–34.9	11 (61.1)
	≥ 35.0	4 (22.2)
Pregestational BMI	≤ 25	13 (72.2)
	25.1–29.9	4 (22.2)
	≥ 30	1 (5.6)
Parity	Nulliparity	1 (5.6)
	Multiparity	17 (94.4)
Any use of tobacco in 1. trimester	Yes	2 (11.1)
	No	16 (88.9)
Timing of sepsis	Post-abortion	1 (5.6)
	During pregnancy	2 (11.1)
	Intrapartum	2 (11.1)
	Postpartum	13 (72.2)
Pathogen in blood culture	No microbe identified	8 (44.4)
	Gram negative rods	6 (33.3)
	Beta-haemolytic streptococci	2 (11.1)
	Blood culture not obtained	1 (5.6)
	Missing	1 (5.6)
Mode of delivery	Post-abortion	1 (5.6)
	Spontaneous vaginal delivery	8 (44.4)
	Assisted vaginal delivery	3 (16.7)
	Elective caesarean delivery	0 (0.0)
	Emergency caesarean delivery	6 (33.3)
Focus of infection	Post-abortion endometritis	1 (5.6)
	Chorioamnionitis	1 (5.6)
	Postpartum endometritis^a^	7 (38.9)
	Perineal wound infection	0 (0.0)
	Caesarean wound infection	0 (0.0)
	Urinary tract infection	
	Antepartum	2 (11.1)
	Postpartum	2 (11.1)
	Unknown focus^b^	5 (27.8)
Source control performed	No	9
	Yes	9
Lowest systolic blood pressure measured in mmHg	≤ 89	3 (16.7)
	90–99	2 (11.1)
	100–139	8 (44.4)
	≥ 140	4 (22.2)
	Missing	1 (5.6)
Lowest diastolic blood pressure registered in mmHg	≤ 40	1 (5.6)
	40–49	2 (11.1)
	50–89	12 (66.7)
	90–99	1 (5.6)
	≥ 100	1 (5.6)
	Missing	1 (5.6)
Highest heart rate registered (min^-1^)	61–99	1 (5.6)
	100–119	4 (22.2)
	≥ 120	13 (72.2)
Highest respiration rate registered (min^-1^)	Nov-19	2 (11.1)
	20–24	6 (33.3)
	≥ 25	6 (33.3)
	Missing	4 (22.2)
AVPU	Alert	18 (100)
	Verbally responsive	18 (100)
	Pain stimulus responsive	18 (100)
	Unresponsive	0 (0.0)
Temperature (◦C)	≤ 36	0 (0.0)
	36.1–37.9	2 (11.1)
	38.0–39.0	6 (33.3)
	≥ 39.1	10 (55.6)
Leukocyte count (10^9^/L)	0–4.0	0 (0.0)
	4.1–12.0	4 (22.2)
	≥ 12.1	13 (72.2)
	Missing	1 (5.6)
Sepsis treatment initiated at time of sepsis diagnosis	Yes	13 (72.2)
	No	5 (27.8)
Initial antibiotic treatment	Benzylpenicillin, gentamicin	5 (27.8)
	Ampicillin, metronidazole	3 (16.7)
	Ampicillin, gentamicin	3 (16.7)
	Benzylpenicillin, clindamycin, gentamicin^c^	2 (11.1)
	Ampicillin, gentamicin, metronidazole	2 (11.1)
	Ampicillin, clindamycin	1 (5.6)
	Metronidazole, gentamicin	1 (5.6)
	Cefotaxime	1 (5.6)
Puerperal sepsis regimen^c^ given in puerperal sepsis (n = 13)	Yes	2 (15.4)
	No	11 (84.6)
Intensive care	Yes	8 (44.4)
	No	10 (55.6)
NICU	Yes	6 (33.3)
	No	11 61.1)
	NA	1 (5.6)
Duration of labour (hours)	0–10	10 55.6)
	≥ 10.1	7 (38.9)
	NA	1 (5.6)
No. of vaginal examinations	≤ 5	10 (55.6)
	≥ 6	7 (38.9)
	NA	1 (5.6)
Induction of labour	Yes	12 (66.7)
	No	5 (27.8)
	NA	1 (5.6)
Ruptured membranes (hours)	≤ 10	13 (72.2)
	≥ 10.1	4 (22.2)
	NA	1 (5.6)
Estimated blood loss (millilitres)	≤ 500	12 (66.7)
	501–1000	3 (16.7)
	≥ 1001	3 (16.7)
Length of hospital stay (days)	≤ 3	5 (27.8)
	4-7	6 (33.3)
	≥ 8	7 (38.9)
Maternal death	Yes	0 (0.0)
	No	18 (100)
Neonatal death	Yes	0 (0.0)
	No	17 (94.4)
	NA	1 (5.6)

*BMI* body mass index, *AVPU* a scale that is useful to rapidly grade a patient’s gross level of consciousness, responsiveness, or mental status, *NICU* neonatal intensive care unit, *NA* not applicable.

^a^2/7 cases of sepsis with endometritis as focus resulted from caesarean section.

^b^3/5 cases of sepsis with unknown origin as focus were associated with caesarean section.

^c^Indicates the antibiotic of choice in the local and national guideline for puerperal sepsis.

A microbial aetiology by culture was identified in 44.4% (of sepsis cases, gram-negative rods (predominantly Enterobactereales) in 33.3% and beta-haemolytic streptococci in 11.1%. In total 72.2% of women were given broad-spectrum antibiotics intravenously at the time of sepsis diagnosis. In only 15.4% of the postpartum cases the antibiotic of choice adhered with the puerperal sepsis guideline. Half of the women were subject to source control with evacuation of an infected focus.

We found that O-codes were most frequently used during the study period and that sepsis patients were recorded among these; unaccompanied by R-codes. The code R65.1 (infection with organ dysfunction) was used in a single case only of postpartum sepsis. An A-code was only applied in a single case, whereas B-codes were applied ten times. Table 5 shows that O85 defined as “Puerperal fever” was the diagnostic code most frequently applied to encode puerperal infection and involves three conditions: postpartum endometritis, peritonitis, and *puerperal sepsis*^20^. The code O85 was correctly applied in the coding of postpartum endometritis with 91% sensitivity and 81% positive predictive value. In the case of puerperal sepsis and maternal sepsis the positive predictive value was 20.3% for both conditions.

**Table 5:** Assessment of the accuracy of diagnostic codes for postpartum endometritis, puerperal sepsis, peritonitis, and maternal sepsis according to the medical records of obstetric patients, January–December 2016, Haukeland University Hospital, Norway.

O85* in total (postpartum endometritis, peritonitis, and puerperal sepsis)				
		Medical records		
		+	−	Total births
Diagnosis of infection	+	62	2	64
	−	6	5112	5118
	Total	68	5114	5182

		Number	Percentage (95% CI)	
Positive predictive value		62/64	96.9 (89.3–99.1)	
Negative predictive value		5112/5118	99.9 (99.7–100)	
Sensitivity		62/68	91.2 (82.1–95.9)	
Specificity		5112/5114	100 (99.9–100)	

O85 Postpartum endometritis				
		Medical records		
		+	−	Total births
Diagnosis of infection	+	52	12	64
	−	5	5113	5118
	Total	57	5125	5182

		Number	Percentage (95% CI)	
Positive predictive value		52/64	81.3 (70.0–88.9)	
Negative predictive value		5113/5118	99.9 (99.8–100)	
Sensitivity		52/57	91.2 (81.1–96.2)	
Specificity		5113/5125	99.8 (99.6–99.9)	

O85 Puerperal sepsis				
		Medical records		
		+	−	Total births
Diagnosis of infection	+	13	51	64
	−	2	5116	5118
	Total	15	5167	5182

		Number	Percentage (95% CI)	
Positive predictive value		13/64	20.3 (12.3–31.7)	
Negative predictive value		5116/5118	99.9 (99.9–100)	
Sensitivity		13/15	86.7 (62.1–96.3)	
Specificity		5116/5167	99.0 (98.7–99.3)	

O85 Peritonitis				
		Medical records		
		+	−	Total births
Diagnosis of infection	+	3	61	64
	−	0	5118	5118
	Total	3	5179	5182

		Number	Percentage (95% CI)	
Positive predictive value		3/64	4.7 (1.6–12.9)	
Negative predictive value		5118/5118	100 (99.9–100)	
Sensitivity		3/3	100 (43.9–100)	
Specificity		5118/5179	98.8 (98.5–99.1)	

Maternal sepsis encoded with O85				
	Medical records			
		+	−	Total births
Diagnosis of infection	+	13	51	64
	−	4	5114	5118
	Total	17	5165	5182

		Number	Percentage (95% CI)	
Positive predictive value		13/64	20.3 (12.3–31.7)	
Negative predictive value		5114/5118	99.9 (99.8–100)	
Sensitivity		13/17	76.5 (52.7–90.5)	
Specificity		5114/5165	99.0 (98.7–99.3)	

*In The International Statistical Classification of Diseases and Health Related Problems, 10th Edition, O85 is defined as “puerperal fever” and represents an infection arising from the birth canal. The code represents postpartum endometritis, peritonitis, and puerperal sepsis. There is no separate code for maternal sepsis in the ICD-10.

## Discussion

In a hospital cohort we found 0.9% infection following legal abortion and 7.8% obstetric infection during pregnancy, in labour and the postpartum period. The incidence of maternal sepsis was 0.3% during the study period. Most cases of maternal sepsis were recorded in the postpartum period. Evaluation of clinical handling in cases of maternal sepsis showed that two thirds of patients were given broad-spectrum antibiotics at the time sepsis was suspected, but only 15.4% of women with puerperal sepsis were given antimicrobials in accordance with national antibiotic guidelines. Assessment of the relevant diagnostic code (O85) for maternal sepsis showed low positive predictive value.

Until recently there were no epidemiological studies on sepsis in Norway. Knoop et al. showed in 2017 that in hospitalised patients, sepsis incidence for the entire Norwegian population was 140 per 100,000, equivalent to 0.14%^25^. Maternal sepsis studies from other parts of the world show an incidence of 0.03% in the USA with a case fatality rate of 4.4 per 100 cases^26^. In Scotland the incidence of maternal sepsis during the years 1987–2008 was found to vary between 0.31 and 2.11%, whilst for septic shock it varied between 0.00 and 0.24%^27^. The Scottish findings were based upon separate ICD-9 codes for septicaemia, sepsis following abortion, puerperal sepsis, and septic shock, respectively. Together these studies illustrate that maternal sepsis is rare, but also that comparison of sepsis incidence between different regions and countries, in addition to different time periods, is challenging. In contrast to Scotland, we have no unique diagnostic code for maternal sepsis in Norway. Our finding of 0.3% is however higher than in the USA but is consistent with that from Scotland.

We identified a single case of maternal sepsis following spontaneous abortion after a pregnancy with cervical cerclage and none following legal abortion in the study period. Our finding of 0.9% occurrence of infection following legal abortion is consistent with the findings in a prospective study from Haukeland University hospital during 2006–2009^28^. All but one of the nine women with post-abortion endometritis following legal abortion, had surgical evacuation within the same day they presented with infection to our hospital, resulting in fast recovery. A Finnish study from 2009 investigated immediate complications after legal abortions (both medical and surgical) and found 0.8% and 0.6% infection with evacuation in the group of medical and surgical abortion groups, respectively^29^. Arguably, it may be that in countries where abortion is legalised, both near-miss cases and fatalities due to septic abortion are rare, as is the case for Norway where infections following legal abortions are treated promptly.

Concurrently, to the best of our knowledge, there are no epidemiological studies on the incidence of maternal sepsis in the Nordic countries. A Swedish study from 2014 used self-reported patient data to give incidence of puerperal infection both with and without prescription of antibiotics. A total occurrence of postpartum infection of 11.5% was found consisting of 4.7% mastitis (2.9% treated with antibiotics), 3.0% urinary tract infection (2.4% treated with antibiotics), 2.0% endometritis (1.7% treated with antibiotics), and 1.8% perineal wound infection (1.2% treated with antibiotics)^30^. However, the cases with infection were not confirmed either through medical records or prescription registries. The occurrence of infection found in that study cannot be applied to estimate the number of patients requiring hospital admission and is therefore not comparable to results from this study. The incidence of antepartum or intrapartum infections was not examined in the Swedish study, neither was maternal sepsis. The same authors examined the incidence of postpartum sepsis by a search for diagnostic codes in the Swedish ICD-10 and based upon this, the prevalence of postpartum sepsis was 2.4/10,000 women. The weakness in that study is that the code O85 is not equivalent to postpartum sepsis as it can be utilized in cases of infection without the presence of organ dysfunction, as we have demonstrated in our study as well^31^. However, sepsis after abortion, in pregnancy and labour were not examined in that study.

Our study indicates that approximately two thirds of patients were given broad spectrum antibiotics at the time sepsis was diagnosed. Concerningly, only two of the thirteen women with puerperal sepsis were given antibiotics in accordance with the Norwegian national guidelines for intrahospital antibiotic treatment, and eight different antibiotic regimens were used. A possible explanation for the apparent low adherence to guidelines could be that sepsis went unrecognized due to a lack of sepsis definition but also insufficient awareness of the condition. This illustrates the need to both examine the compliance of guidelines in cases of maternal sepsis as well as raise awareness of the condition.

Obstetric infections and maternal sepsis have suffered neglect to be recognized as important areas of maternal health. There are several explanations for this; firstly, all normal physiologic parameters of pregnancy overlap with those of the former Systemic Inflammatory Response Syndrome (SIRS) criteria^10^, which provide low specificity for the identification of sepsis in all patient categories^32^. Secondly, the contemporary sepsis definition for the general population excludes the obstetric population^8^. Finally, no definition of obstetric sepsis has so far been applicable to all stages of pregnancy, i.e., ante-, intra- and postpartum periods.

Delimiting maternal sepsis is difficult. As this study illustrates most obstetric infections are associated with the postpartum period. Postpartum endometritis is indeed the commonest focus of infection leading to maternal sepsis. Only the cases with organ dysfunction are however classified as maternal sepsis. This distinction is crucial, and the recently established maternal sepsis definition by the WHO may lead to more precise recording. Additionally, the new definition may spur further studies that will lead to better understanding of the condition across institutions and countries.

Audits on maternal deaths in the United Kingdom (UK), the MBRRACE reports, demonstrating that maternal sepsis arising from the genital tract was the leading cause of maternal morbidity during the years 2006–2008, have led to increased focus on maternal infection in the recent years^4^. Also, it was concluded that more than half of the sepsis related maternal deaths were preventable, which has given rise to several quality improvement efforts in maternal health care in the UK. One of the major efforts is the development of modified early warning score systems for the obstetric population to detect clinical deterioration in cases of infectious morbidity.

There was no practice of systematic or periodic scoring of physiologic parameters (e.g., *“early warning score systems”*) for admitted nor outpatient obstetric patients in the study period at our hospital. Almost all the 18 women with maternal sepsis had an aberration in one or several physiologic parameters: tachycardia, pyrexia, and tachypnoea occurring most frequently. The use of vital parameters shows favourable in detecting deterioration from infection in the obstetric patient. Such systems are established for 30 years in the general adult population^33,34^. However, the use of scoring systems is not yet evaluated for the obstetric population and needs further investigation^35^. We implemented a scoring system for obstetric patients in 2018 in obstetric departments in Norway, to standardize the use of vital parameters for uncovering of clinical deterioration^36^. During the covid-19 pandemic we learned that obstetric patients proved to be an extra vulnerable population for serious morbidity and mortality^37^. Proper use of scoring systems makes us better prepared to meet future challenges.

The diagnostic code most frequently applied to encode puerperal sepsis in the study period, was O85, *puerperal fever * or *“child bed fever”*. However, this diagnostic code also comprises postpartum endometritis and peritonitis. Its positive predictive value for puerperal and maternal sepsis makes it less useful for the exploration of the true incidence of maternal sepsis in a retrospective manner. Considering that this code was accompanied by an R-code only once during the study period, the indication of organ dysfunction was lacking in most cases. Consequently, it cannot be applied for surveillance purposes of maternal sepsis in Norway. For sepsis occurring antepartum, there exists no specific code. Only intrapartum sepsis has its own diagnostic code, O75.3, “sepsis in labour”. However, this code was only applied once during the year 2016 at HUH. Hence, there is no specific sepsis code for the obstetric population which encompasses the entire pregnancy in the Norwegian ICD-10, but neither exists a unique diagnostic code for antepartum or postpartum sepsis^20^.

The present study had several limitations. The retrospective design provided limited information to the administration of prophylactic antibiotics during labour or caesarean delivery. According to our hospital guideline, all women undergoing emergency caesarean section are given prophylactic antibiotics, while normal weight, healthy nulliparous women are not given antibiotics. However, we have in a previous study showed that elective caesarean sections do not contribute to maternal infectious morbidity, and that prophylactic antibiotics should be restrictedly given, upon indication only, to women undergoing elective caesarean section^38^. Six of the patients with maternal sepsis in our study were delivered by emergency caesarean section, accounting for 33.3% of the sepsis cohort. Some of the sepsis cases were not scored according to respiration rate, indicating that this parameter was not systematically measured in 2016. Additionally, the retrospective design limited information to the timing of antibiotic treatment.

Another limitation is that we could have missed several cases of less severe infections with for instance endometritis and mastitis diagnosed and handled in the primary health care, which is highly available in Norway. Also, patients admitted to other departments outside the maternity department, were not included. Incidental infections other than urinary tract infections, for instance respiratory tract infections, were hence not recorded as they are not registered with O-codes. Furthermore, we could have missed some patients with severe infection if other diagnostic codes were used, regardless of searching for hospital stays lasting seven days or longer. In 2016 the coding practice shows that O-codes were applied unaccompanied by R-codes and A-codes, resulting in lacking sepsis indication. B-codes which specify bacterial aetiologies but without sepsis indication, were however applied ten times during 2016, further indicating that the coding practice for obstetric sepsis was not in accordance with the national coding instructions. Our incidence data might therefore be lower than the actual incidence.

This study does not provide the incidence of infections after spontaneous abortion as the denominator of spontaneous abortion is unexplored.

The strength of this study was the inclusion of a great number of obstetric infections according to the ICD-10 and a review of the medical records by an expert council to ensure that the cases with infection were correctly coded. All sepsis cases met the diagnostic criteria according to the WHO. Our findings on the occurrence of obstetric infections other than sepsis are most probably applicable to other Norwegian and Nordic maternity departments.

## Conclusions

In conclusion, in a Norwegian hospital cohort of pregnant, labouring, and postpartum women obstetric infections were frequent. Most obstetric infections occurred in the postpartum period, as did maternal sepsis. Evaluation of the management in sepsis cases showed that there was low adherence to national antibiotic guidelines in cases of puerperal sepsis. The study concludes that coding of maternal sepsis is poor, as there exists no separate code for this condition. We suggest that a specific diagnostic code for maternal sepsis is constructed to offer more accurate diagnosis, increasing coding compliance and improving surveillance at regional and national level. We plan to do a follow-up study to investigate how implementation of an obstetrically modified early warning score system in our hospital has affected the diagnostics and handling of maternal sepsis.

### Supplementary Information

Supplementary Information.

## Data Availability

The datasets used and analysed during the current study are available from the corresponding author on reasonable request.
