# Supplementary material for: Obstetric infections and clinical characteristics of maternal sepsis: a hospital-based retrospective cohort study
Source: Sci Rep. 2024 Mar 13;14:6067. doi: 10.1038/s41598-024-56486-4 (PMC10937963; doi:10.1038/s41598-024-56486-4)
Supplement: Supplementary file 1 — Supplementary Information. [file 41598_2024_56486_MOESM1_ESM.docx]

**Supplementary information to “****Obstetric infections and clinical characteristics of maternal sepsis. A hospital-based retrospective cohort study.”**

*Sedina Atic Kvalvik^1,2^, Sofie Branæs Zakariassen^2#^, Sofie Overrein^2#^, Svein Rasmussen^2^, Steinar Skrede^2,3^, Elham Baghestan^1,2^*

Department of Obstetrics and Gynaecology, Haukeland University Hospital, Pb 1400, N-5021 Bergen, Norway

Department of Clinical Science, University of Bergen, Pb 7804, N-5020 Bergen, Norway.

Department of Medicine, Haukeland University Hospital, Pb 1400, N-5021 Bergen, Norway

# Equal contributions to the study

Submitted to Scientific Reports

Corresponding author: Sedina Atic Kvalvik

ORCHID ID: <https://orcid.org/0000-0001-9364-1816>

Address: Department of Clinical Science, University of Bergen, Pb 7804, N-5020 Bergen, Norway

Telephone: + 47 95 15 29 71

E-mail address: sedina.atic.kvalvik@helse-bergen.no

**Table S1** Overview of the diagnostic codes examined in the study.

| **Diagnostic code in the ICD-10**^a^ | **Definition of code** |
| --- | --- |
| A40.0 | Group A streptococcal sepsis |
| A40.1 | Group B streptococcal sepsis |
| A40.2 | Enterococcal sepsis |
| A40.8 | Other specified streptococcal sepsis |
| A40.9 | Not otherwise specified streptococcal sepsis |
| A41 (.0-2) | Staphylococcal sepsis |
| A41.3 | Hemophilus influenzae A sepsis |
| A41.4 | Sepsis with anaerobic microbes |
| A41.5 | Sepsis with gram negative microbes |
| A41.8 | Other specified sepsis |
| A41.9 | Not otherwise specified sepsis |
| A48.3 | Toxic shock syndrome |
| B95 (.0-8) | Streptococcal and staphylococcal infection |
| B96 (.0-8) | Other specified bacterial infection |
| O02.0 | Incomplete abnormal pregnancy, complicated by infection |
| O02.5 | Complete abnormal pregnancy, complicated by infection |
| O03.0 | Incomplete spontaneous abortion, complicated by infection |
| O03.5 | Complete spontaneous abortion, complicated by infection |
| O04.0 | Incomplete legal abortion, complicated by infection |
| O04.5 | Complete legal abortion, complicated by infection |
| O07.0 | Failed attempt of legal abortion, complicated by infection |
| O07.5 | Other failed attempts of legal abortion, complicated by infection |
| O08.0 | Infection following abortion and extrauterine pregnancy |
| O23.0 | Urinary tract infection during pregnancy |
| O41.1 | Chorioamnionitis |
| O75.1 | Shock during labour or delivery |
| O75.2 | Fever in labour, not otherwise classified |
| O75.3 | Other infection during labour (includes sepsis but excludes chorioamnionitis) |
| O85 | Puerperal fever (includes postpartum endometritis, puerperal sepsis, and peritonitis) |
| O86.0 | Infection in obstetric wound (includes perineal and abdominal wounds) |
| O86.1 | Other genital tract infection following delivery |
| O86.2 | Urinary tract infection following delivery |
| O86.3 | Other infections in the urinary or genital tract following delivery |
| O86.4 | Pyrexia from unknown origin following delivery |
| O86.8 | Other specified puerperal infection |
| O90.0 | Wound rupture after caesarean delivery |
| O90.1 | Perineal wound rupture |
| O90.2 | Obstetric wound hematoma (includes perineal and abdominal wounds) |
| O91.1 | Abscess of the breast in pregnancy or following delivery |
| O91.2 | Non-purulent mastitis in pregnancy or following delivery |
| O98.8 | Other specified infectious diseases which complicate pregnancy, labour, and postpartum period |
| R57.2^b^ | Septic shock |
| R57.8^b^ | Other specific shock caused by endotoxins |
| R65.1^b^ | Systemic inflammatory response syndrome caused by infection with organ dysfunction |

^a^ ICD-10, The International Statistical Classification of Diseases and Related Health Problems, 10^th^ Edition

^b^ Obstetric sepsis in accordance with the Norwegian coding instruction consists of an O-code indicating an obstetric infectious condition accompanied by an R-code to indicate organ dysfunction or septic shock, alternatively an A-code instead of R-code to indicate bacterial aetiology in sepsis cases. B-codes are used to identify the bacterial agent, but do not indicate sepsis and should therefore accompany O- and R-codes in sepsis cases.
